# Bio-driven visual saliency detection with color factor

**DOI:** 10.3389/fbioe.2022.946084

**Published:** 2022-08-04

**Authors:** Yan Wang, Teng Li, Jun Wu, Chris H. Q. Ding

**Affiliations:** ^1^ School of Computer Science and Technology, Anhui University, Hefei, China; ^2^ School of artificial intelligence, Anhui University, Hefei, China; ^3^ Guangdong CAS Cogniser Information Technology Co., Ltd., Guangzhou, China; ^4^ School of Data Science, The Chinese University of Hong Kong, Shenzhen, China

**Keywords:** bio-driven, color space, saliency detection, fixation prediction, human attention

## Abstract

Most visual saliency computing methods build models based on the content of an image without considering the colorized effects. Biologically, human attention can be significantly influenced by color. This study firstly investigates the sole contribution of colors in visual saliency and then proposes a bio-driven saliency detection method with a color factor. To study the color saliency despite the contents, an eye-tracking dataset containing color images and gray-scale images of the same content is proposed, collected from 18 subjects. The CIELab color space was selected to conduct extensive analysis to identify the contribution of colors in guiding visual attention. Based on the observations that some particular colors and combinations of color blocks can attract much attention than others, the influence of colors on visual saliency is represented computationally. Incorporating the color factor, a novel saliency detection model is proposed to model the human color perception prioritization, and a deep neural network model is proposed for eye fixation prediction. Experiments validate that the proposed bio-driven saliency detection models make substantial improvements in finding informative content, and they benefit the detection of salient objects which are close to human visual attention in natural scenes.

## 1 Introduction

When viewing a visual scene, the human visual system can quickly focus on some unique vision areas. An understanding of human biological mechanisms in visual saliency detection is essential to many applications, including video segmentation (Ren et al., 2021), target detection Chong et al. (2020), image enhancement Sun et al. (2022), and activity recognition Jiang et al. (2019), Chen et al. (2021).

**FIGURE 1:**
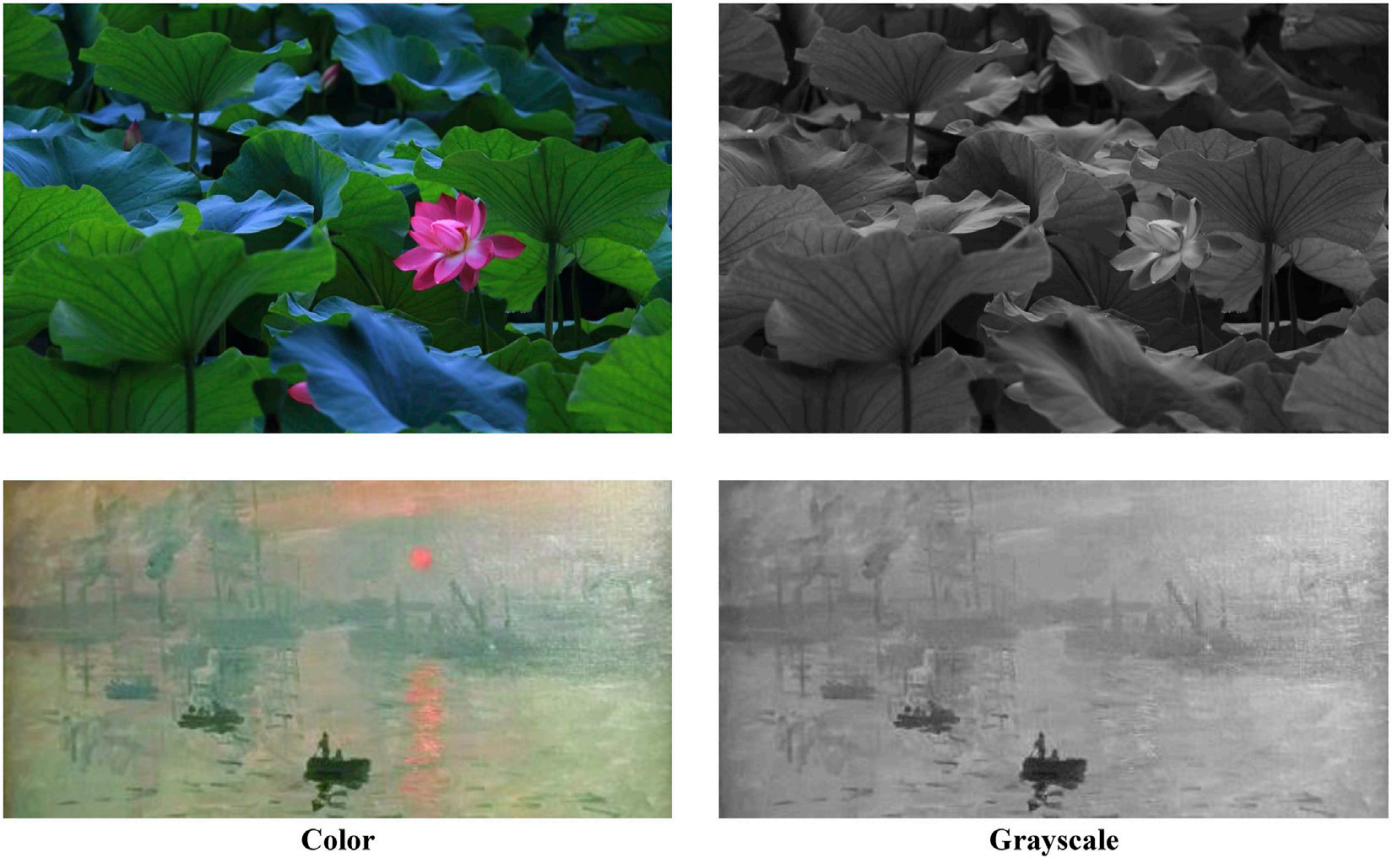
Visual comparisons of color images and gray-scale images of the same content. In the images in color mode, objects (lotus and sun) exhibit a pop-out effect due to their distinguishable color, while in gray-scale mode, the guiding power of these objects is almost the same as the rest of the surroundings, and this information has little effect on visual attention.

Saliency in a visual scene can arise from a spectrum of stimuli, both low-level image properties and semantic-level information Rosenholtz et al. (2011). In human visual system, color, besides contrast, intensity, and motion, is considered one of the primary features in computing bottom-up saliency. As we can see from the example in Figure 1, the guiding powers of color stimuli and grayscale stimuli are vastly different for visual attention. By comparing the eye fixation maps in both color images and gray-scale images of the same content, it is clear that color has its sole contribution to visual saliency. However, many existing attention models usually neglect the colorized effects and predict the same results for images containing the same content. The saliency based solely on color has not been well studied. One reason is the lack of eye tracking datasets including color images and gray-scale images of the same content.

However, previous visual saliency models considering color attributes address a problem that is relatively ill-posed. Classical saliency models Gelasca et al. (2005), Choi and Suk (2015) evaluate which colors attract more attention based on a subjective experiment, considering only a few colors, and the results of these studies cannot be extended to natural visual scenes. Moreover, these studies did not consider the effects of content information (e.g., position/order) rather than color. Researchers have not yet attempted to computationally model the relationship between visual attention and color, despite its contents.

In this paper, we claim that the computational model should consider the saliency of the image separately in color and gray-scale scenes and propose a new Color-Gray eye-tracking dataset (CGed) focusing on image color and human attention. Statistical analysis on CGed is conducted to investigate how colors influence human attention when viewing natural scene, how much the colors attract human attention, and how to model the factor of colors in visual saliency computing. Analytical results indicate that certain colors attract human visual attention strongly, and the color component b of the CIELab color space is closely related to visual attention more closely than the others—which we refer to as the color prioritization effect.

Based on these discoveries, we propose a salient object detection model RNCw (Region contrast based on Non-uniform quantification and Channel-weighted color distance) by incorporating the color prioritization effect into the previous method proposed by Cheng et al. (2015). We further apply our discovery in eye fixation prediction and design a color weighted DNN (APNet—Not Adaptive color weighting priori attention weighting Network) model. Experiments demonstrate the superior performance of the models we proposed, especially when color-eliciting objects stand out in a scene. Our contributions can be summarized as follows:

1) We propose a new image dataset (CGed) featuring visual attention. To the best of our knowledge, this is the first dataset that contains both color images and grayscale images of the same content. It is designed for research on visual saliency, especially with regard to the effect of image color on saliency despite image content.
2) We evaluate how colors attract human attention computationally. We observe that some particular colors attract our attention more than others, and that certain combinations of color blocks can enhance attention.
3) We proposed two novel saliency computational models incorporating the color factor: RNCw model, which is compliant with human perception to improve the performance in detecting salient objects; and APNet model that encodes relative importance of objects in an image to achieve predictions more coinciding with human visual attention. The proposed models achieve state-of-the-art performance on benchmark datasets.

## 2 Related works

### 2.1 Color and visual saliency model

Since color information plays an important role in visual attention biologically, it has been used in saliency computation in previous works. Osberger Pappas (2001) suggested that some particular colors (e.g., red) attract our attention more than others, or induce a higher amount of masking. However, saliency researchers have not yet investigated what color attracts human attention despite its content when viewing natural scenes. One major reason could be the lack of a proper dataset with both color images and gray-scale images of the same content.

Achanta et al. (2008), Achanta and Süsstrunk (2010) use the color and luminance features to detect salient objects. They calculated the contrast between the local image region and its surroundings. The saliency map can be obtained by calculating the average color vector difference. Borji and Itti (2012) propose a prediction model to reflect the saliency discrimination towards eye tracking data. The model measures the scarcity of each block in both RGB and LAB color space, and then combines the local and global saliency of each color space to generate the saliency map. As stated above, many saliency models compute image saliency primarily by measuring the color feature. However, these models did not clearly consider the sole contribution of colors, excluding the image content factor.

In the past decade, substantial research has been done on visual attention computational models to predict saliency. Traditional attention models mainly rely on various cues to detect salient objects, including local contrast Klein and Frintrop (2011), global contrast Cheng et al. (2011) and background prior Yang et al. (2013). Subsequent behavioral and computational studies started to predict fixation with saliency maps to understand human visual attention and verify saliency models. A large gain in saliency prediction has resulted from the recent resurgence of convolutional neural networks (CNNs). Specifically, several methods such as Liu et al. (2015) used CNN to extract features from multiple images region with varying contexts, and then combined these contextual features to infer saliency. Some other models, such as Li and Yu (2016), adopt fully convolutional networks (FCNs) for feature representation at each image location and generate saliency maps in a convolutional way. Recent developed visual representation models such as the visual transformer have also been applied to salient object detection Liu et al. (2021), and they achieved high performance on previous datasets.

Along with these advances, attention models can effectively extract visual features and compute feature maps to quantify saliency. However, existing methods did not consider the unique influence of colors in saliency computing models, while human visual attention order is sensitive to different colors in a natural scene. By weighting the contribution of color to attention, our work effectively addresses the color prioritization effect on attention allocation in an image.

### 2.2 Eye-tracking datasets

Several datasets have been introduced to further challenge the eye fixation prediction model. Two widely-used image datasets are the MIT dataset Judd et al. (2010), which contains 1,003 natural images free-viewed by 15 subjects each, and the NUSEF dataset Ramanathan et al. (2010), which includes 758 (emotion evoking) images free-viewed by 25 subjects each. There are other datasets focusing on specific domains: OSIE Xu et al. (2014) features multiple dominant objects in each image, and CAT2000’s training set contains 2,000 images of diverse scenes, such as affective images and cartoons. However, there are few eye-tracking datasets suitable for research regarding the saliency of color despite its content. In this paper, we present the first eye-tracking dataset to include both color images and grayscale images of the same content.

## 3 Construction of the CGed dataset

The saliency of images considering content has been extensively explored, but the saliency based solely on color has not received much attention, probably due to the lack of eye-tracking datasets including color images and gray-scale images of the same content. To address the problem, we constructed CGed, a new dataset containing both color images and gray-scale images of the same content, with eye-tracking data. It is designed for research on visual saliency, especially with regard to the saliency of color, despite its content.

### 3.1 Image collection

CGed images were collected partially from the MIT1003 dataset and partially from an online image search engine. It contains a total of 500 brightly colored images with various semantic concepts ranging from rural to urban environments. These 500 images of natural scenes are rich in color. All color images were then converted to grayscale, so that the CGed dataset includes a total of 1,000 images, with 500 grayscale images. We collected the images to make the dataset more diverse regarding how observers’ attention is attracted.

### 3.2 Eye tracking

Eighteen subjects freely observe all CGed images on a 22-inch LCD monitor for 5 s. Nine of the subjects are male and the others are female, and their ages are distributed in the range of 22 to 29. These subjects can focus their attention on given images and yield precise annotation. The screen resolution of an LCD monitor is 1,680, ×, 1,050. The visual angle of the stimuli is about 42.48° × 27.31°. Eye movements of the subjects are recorded using the SensoMotoric Instruments (SMI) iView X RED system. Eye position is recorded with an eye tracker operating at a 250 Hz sample rate.

## 4 Computational studies of color factor on visual saliency

In this section, we study the contribution of colors despite content to attention when viewing natural scenes. We first explain our analytical methods and then report observations with supporting analyses.

A necessary prerequisite for showing an influence the color on attention is the difference in attention score between the color image and the corresponding grayscale image. Comparing attention scores between color images and gray-scale images of the same content reveals a general influence of color on visual attention since all other image features remain the same. We first study the contribution of color to attention. Based on the finding of salient colors, we study the influence of combination of color blocks to attention.

### 4.1 Definitions and methods

For the study on CGed, we used a common method in saliency research Le meur and Baccino (2013). Specifically, for each image, we compute a fixation map by placing at each fixation location a Gaussian distribution with sigma equal to one degree of visual angle, and then normalizing the map to maximum 1. We define the attention score of an image pixel as the fixation-map value at this pixel. The attention score of each pixel thus ranges between 0 and 1. In order to study the effect of color on attention, we compute the difference in attention score (DAS) by subtracting the attention score of the color image from the attention score of the gray-scale image.

We use the Maximal Information Coefficient (MIC) David et al. (2011) to analyze the correlation between color and attention. MIC is a correlation statistic that measures the association strength of linear and non-linear relationships between paired variables.

$$\begin{array}{cc} MIC & =max\left\{I\left(x,y\right)/{log}_{2}min\left\{n_{x},n_{y}\right\}\right\},where\\ I\left(x,y\right) & =H\left(x\right)+H\left(y\right)-H\left(x,y\right)\\ & =\overset{n_{x}}{\underset{i=1}{\sum }}p\left(x_{i}\right){log}_{2}\frac{1}{p\left(x_{i}\right)}+\overset{n_{y}}{\underset{j=1}{\sum }}p\left(y_{j}\right){log}_{2}\frac{1}{p\left(y_{i}\right)}-\overset{n_{x}}{\underset{i=1}{\sum }}\overset{n_{y}}{\underset{j=1}{\sum }}p\left(x_{i},y_{i}\right){log}_{2}\frac{1}{p\left(x_{i},y_{j}\right)} \end{array},$$ (1)

*n*_*x*_ ⋅ *n*_*y*_ < *B*(*n*), where *B*(*n*) = *n* ^0.6^ is the search-grid size. In calculating MIC for vectors *x* and *y*, *n* is the number of data points, and *n*_*x*_, *n*_*y*_ is the number of bin of partition of the *x* − and *y* − *axis*, respectively. *H*(*x*) and *H*(*y*) represent the entropy associated with *x* and *y*, respectively. And the join entropy of a pair of random variables *x* and *y* is represented as *H* (*x*, *y*).

### 4.2 Statistical results

Which color space is closely related to visual saliency: The color spaces, including RGB, CIELab, and HSI, have been widely adopted by previous studies; the color space has a significant influence on the algorithm performance. In our study, we determined the color space used for data analysis by comparing the correlation between color and saliency in different color spaces.

**FIGURE 2:**
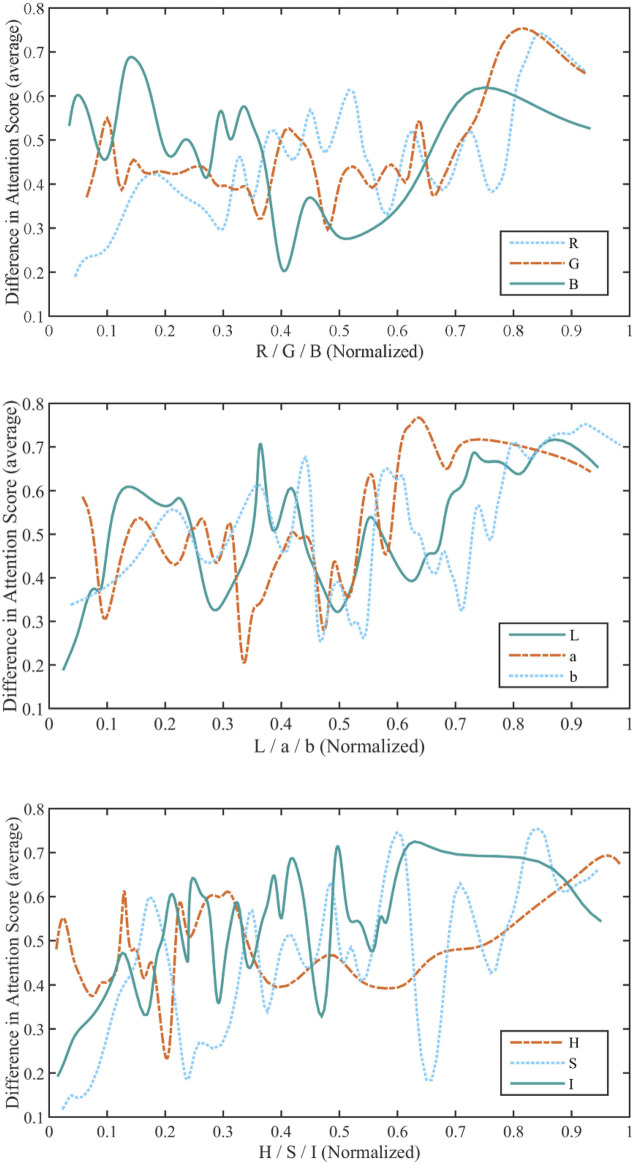
A statistic of the attention score of each color component of the RGB, CIELab, and HSI color spaces for colors corresponding to a DAS greater than 0.1. In the three color spaces, each component of color is normalized to [0,1]. And the MIC between each component and the ADS is calculated (MIC (R,DAS) = 0.57, MIC (G,DAS) = 0.42, and MIC (B,DAS) = 0.36; MIC (L,DAS) = 0.61, MIC (a,DAS) = 0.52, and MIC (b,DAS) = 0.76); MIC (H,DAS) = 0.37, MIC (S,DAS) = 0.41, and MIC (I,DAS) = 0.38).

In our study, only the colors corresponding to a DAS greater than 0.1 are considered salient colors, and the relationship between these colors and attention is studied. We first get a series of colors on the color stimuli, which corresponds to a DAS greater than 0.1. We decomposed the colors in the RGB, CIELab, and HSI color spaces separately into three components for statistical analysis. Since the range of values of color components in the RGB, CIELab, and HSI color spaces are different, we normalized each component. The statistical results are reported in Figure 2. The larger the value of the MIC, the stronger the correlation. The larger total MIC of three color components in CIELab color space over the total MIC of three color components in other color spaces (1.89 *vs.* 1.35 *vs*. 1.16) suggests that saliency is more relevant to CIELab color space than both RGB and HSI color spaces. Subsequent analysis of what color attracts attention, was performed in CIELab color space.

**FIGURE 3:**
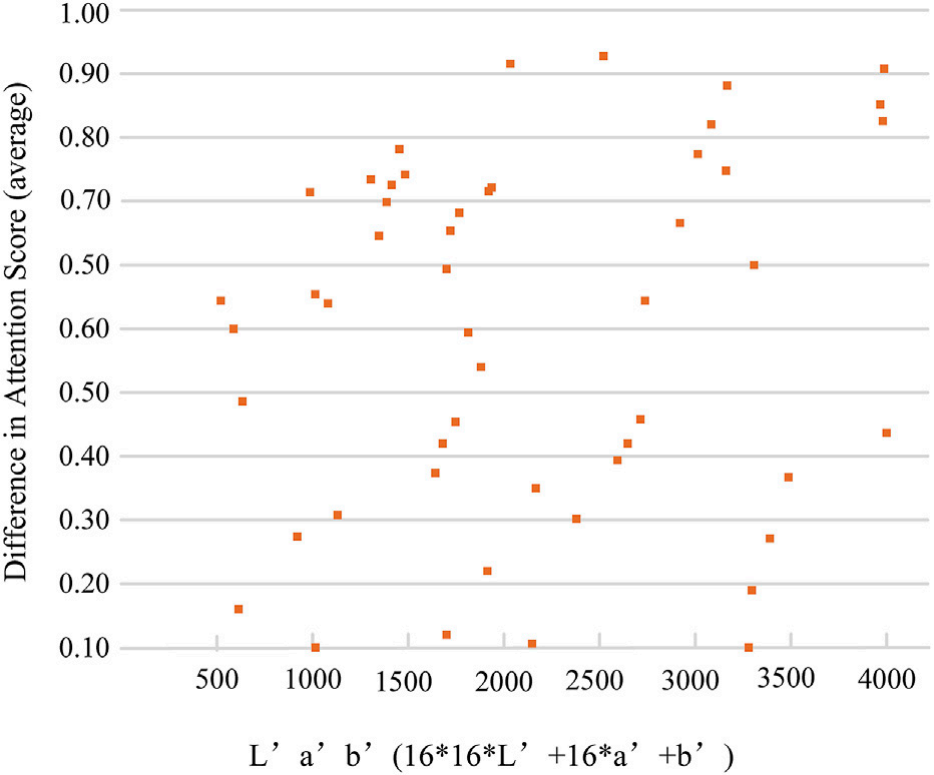
Statistics on the colors that affect attention. Coded colors and the DAS. In CIELab color space, we let L’ = 2.55 *L, a’ = a +127, b’ = b + 127, and code L’a’b’ = 16 * 16 *L’ +16 * a’ + b’.

How colors contribute to attention: We focus on the role of colors in visual attention and try to understand what colors influence more visual attention. We uniformly quantize the value of each color component in the CIELab color space to a range of 0–15, for 16 × 16 × 16 = 4096. The quantized CIELab color space is called the L’a’b’ color space. To see what colors attract attention more intuitively, we encode each color in the L’a’b’ color space. We can see a series of colors and their corresponding attention scores in Figure 3. We also counted ten of the most attractive colors in the CIELAB space. It is noticed that some specific colors have much more saliency. By calculating the difference in the attention score of the color block combination corresponding to the color image and the same content gray-scale image, we also discovered that certain color block combinations often appear with high DAS.

## 5 Proposed color-aware saliency models

In this section, we design our saliency computational models guided by the psychophysics findings in the previous section. On one hand, we propose a salient object detection model RNCw (Region contrast based on Non-uniform quantification and Channel-weighted color distance) to detect salient object/regions. This salient object detection method is an improvement on the RC method Cheng et al. (2015). On the other hand, we designed a color-weighted DNN (APNet—Not Adaptive color weighting priori attention weighting network) for fixation prediction.

### 5.1 RNCw method

The proposed RNCw considers the visual sensitivity of human eyes to different colors in a natural scene, which is a variant of the RC method. The RC method mainly consists of two stages. In the RC method, the input image is first segmented into regions using a graphics-based image segmentation method Felzenszwalb and Huttenlocher (2004), then the color contrast is computed at the region level. The saliency for each region is defined as the weighted sum of the region’s contrast to all other regions in the image. Unlike the RC method, we consider the psychopysics findings in Section 4 in both phases.

In the first phase, different from the RC method, we use the weighted color distance to measure the similarity between pixels in CIELab color space and obtain a segmentation graph. The weighted color distance between the pixels *i* and *j* in an image can be computed as follows:

$$\sqrt{W_{L}{\left(L_{i}-L_{j}\right)}^{2}+W_{a}{\left(a_{i}-a_{j}\right)}^{2}+W_{b}{\left(b_{i}-b_{j}\right)}^{2}}$$ (2)

where *L*_*i*_, *a*_*i*_, and *b*_*i*_ respectively represent the value of pixel *I*_*i*_ in CIELab channels, and *L*_*j*_, *a*_*j*_, and *b*_*j*_ respectively represent the value of pixel *I*_*j*_ in CIELab channels. The *W*_*L*_, *W*_*a,*_ and *W*_*b*_ denote the weights of *L*, *a,* and *b* channels in *CIELab* color space, respectively. The weights *W*_*L*_, *W*_*a,*_ and *W*_*b*_ are determined by correlation coefficient values between attention with the L component, the a component, and the b component in CIELab color space, respectively. Guided by our psychophysics findings, we set *W*_*L*_ = 0.61/1.89 = 0.32, *W*_*a*_ = 0.52/1.89 = 0.28, and *W*_*b*_ = 0.76/1.89 = 0.40. There is no difference in other steps. For more details, refer to Felzenszwalb and Huttenlocher (2004).

In the second phase, we incorporate channel-weighted color distance into the contrast to compute saliency. For a region *r*_*x*_, we compute its saliency value as

$$\begin{array}{c} S\left(r_{x}\right)=\underset{r_{y}\neq r_{x}}{\sum }w\left(r_{x}\right)D_{Wr}\left(r_{x},r_{y}\right) \end{array},$$ (3)

where $w\left(r_{x}\right)$ is the weight of region *r*_*x*_, and the meaning is the number of pixels in the *r*_*x*_ region. $D_{Wr}\left(r_{x},r_{y}\right)$ is the channel-weighted color distance metric between two regions and can be expressed as

$$\begin{array}{c} D_{Wr}\left(r_{x},r_{y}\right)=\overset{n_{1}}{\underset{i=1}{\sum }}\overset{n_{2}}{\underset{j=1}{\sum }}f\left(c_{x},I\right)f\left(c_{y},j\right)D_{W}\left(c_{x,i},c_{y,j}\right) \end{array},$$ (4)

where $f\left(c_{x,i}\right)$ represents the occurrence frequency of the *i* ^*th*^ color in region *r*_*x*_, and $D_{W}\left(c_{x,i},c_{y,j}\right)$ is defined as

$$\begin{array}{c} D_{W}\left(c_{x,i},c_{y,j}\right)=\sqrt{W_{L}{\left(L_{x,i}-L_{y,j}\right)}^{2}+W_{a}{\left(a_{x,i}-a_{y,j}\right)}^{2}+W_{b}{\left(b_{x,i}-b_{y,j}\right)}^{2}} \end{array},$$ (5)

where *L*_*i*,*j*_, *a*_*i*,*j,*_ and *b*_*i*,*j*_ denote the value in *L*, *a*, and *b* channels of the *j* ^*th*^ in region *r*_*i*_, respectively. The *W*_*L*_, *W*_*a,*_ and *W*_*b*_ denote the weights of *L*, *a,* and *b* channels in *CIELab* color space, respectively, whose values are equal to the values in the first phase.

Similar to the approach suggested in the RC method to increase the effects of closer regions and decrease the effects of farther regions, we also incorporate the spatial weighting in terms of Rutishauser et al. (2004). Thus, for any region *r*_*x*_, the saliency is

$$S\left(r_{x}\right)=w_{s}\left(r_{x}\right)\underset{r_{x}\neq r_{y}}{\sum }e^{-\frac{D_{s}\left(r_{x},r_{y}\right)}{\sigma_{s}^{2}}}w\left(r_{y}\right)D_{Wr}\left(r_{x},r_{y}\right),$$ (6)

where $w_{s}\left(r_{y}\right)$ denotes the weight of region *r*_*y*_ which is the number of pixels in region *r*_*y*_, and $D_{s}\left(r_{x},r_{y}\right)$ denotes the spatial distance between the regions *r*_*x*_ and *r*_*y*_. The *σ*_*s*_ controls the strength of spatial distance weighting. As with the RC method, we use $\sigma_{s}^{2}=0.4$ with pixel coordinates normalized to [0,1].

### 5.2 APNet model

**FIGURE 4:**
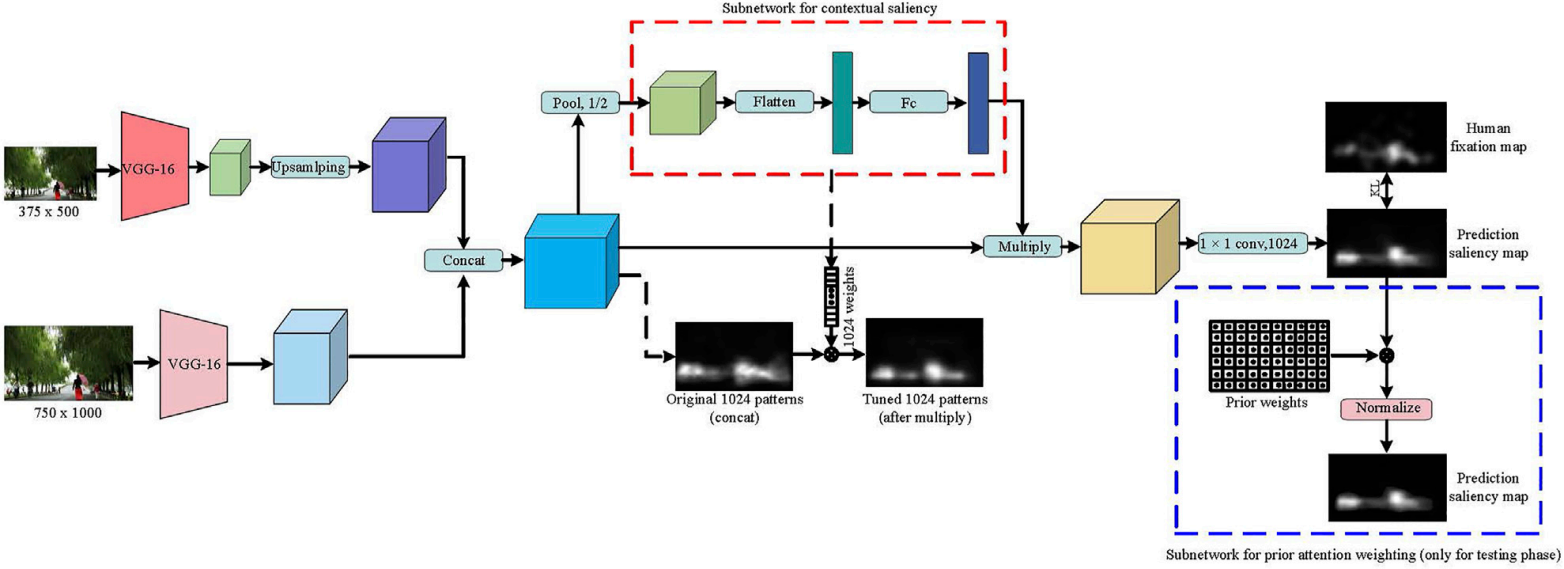
The architecture of the proposed APNet. Two-stream consists of two VGG-16 models which operate on coarse and fine-grained scales of an images. To capture the relative importance of the semantic features of a particular image, a channel-weighted subnetwork (inside the dashed red rectangle) was designed to compute a set of 1024-dimensional features for each image. The priori attention weighting subnetwork (the blue dashed rectangle) directly weights the prediction saliency map output by the DNN, which weights the combines the human eye’s perception sensitivity to color, and can achieve a saliency prediction closer to human visual attention.

We proposed a DNN architecture (APNet—Adaptive color weighting and priori attention weighting Network) is shown in Figure 4. To address color prioritization, we designed a channel weighting subnetwork and *a priori* attention weighting subnetwork. The channel weighting subnetwork (the red dashed rectangle) encodes contextual information, enabling the network to highlight color-eliciting objects from the surroundings. The priori attention weighting subnetwork (the blue dashed rectangle) directly weights the prediction saliency map output by the DNN, which weights the combines the human eye’s perception sensitivity to color, and can achieve a saliency prediction closer to human visual attention. Also, since selective attention may happen at different resolutions, we incorporate information at multiple-scales. The two-stream network design is based on SALICON Huang et al. (2015) and is used to extract deep features from coarse-scale images and fine-scale images.

We briefly introduce our method for using DNN in fixation prediction. We feed fine-scale images of 1,000 × 750 × 3 pixels to its first stream for extracting relatively high-resolution deep features, while feeding coarser-scale images of 500 × 375 × 3 pixels to its second stream for extracting relatively low-resolution deep features. The outputs of the two network streams are rescaled to the same spatial resolution and stacked together to form a multi-scale depth feature of size 32 × 24 × 1024. After applying a 2 × 2 max polling on 1,024 channels of concatenated feature maps to reduce their dimensionality and spatial variance, the channel weighting subnetwork computes a set of 1024-dimensional feature weights for each image. And the weights are applied to the input feature by a channel-wise multiplication. We then performed a convolutional layer after a new subnet with a 1 × 1 kernel to reduce the 1,024 channel 2D image to a single channel 2D saliency map of size 32 × 24 pixels. Lastly, we rescale the saliency map back to the dimension of the original image. In the test phase, each pixel of the output saliency map is multiplied by a weight. Specifically, each pixel in the output saliency map is multiplied by the attention score of each pixel in the original image in the L’a’b color space, and then the saliency map is normalized to a maximum value that is equal to the maximum gray value of the unweighted saliency map. Since the “saliency color” and its attention score in the fourth section are under the condition that the DAS is greater than 0.1, the weight of the “non-saliency color” in the original image is set to 0.1.

## 6 Experiments

We evaluate our salient object detection models on the ECSSD dataset and PASCAL-S within images from a wide-variety of scenarios and resolutions. To demonstrate the effectiveness of the proposed eye fixation prediction model in predicting eye fixations, we evaluated it in CGed, CAT2000, and OSIE datasets.

### 6.1 Salient object detection

#### 6.1.1 Datasets

We test the RNCw model on the ECSSD dataset and the PASCAL-S dataset. ECSSD contains 1,000 structurally complex images acquired from the Internet, and the groundtruth masks were annotated by five labelers. PASCAL-S contains 850 natural images with both saliency segmentation groundtruth and eye fixation groundtruth. Saliency groundtruth masks of PASCAL-S were labeled by 12 subjects.

#### 6.1.2 Evaluation metrics

There are a plethora of metrics available that are used to evaluate saliency models. We use three universally agreed-upon and standard measures for evaluating salient object detection models in salient object detection datasets. They are MAE Borji et al. (2015), S-measure Fan et al. (2017), and Precion-recall (PR) curve Tong et al. (2014).

#### 6.1.3 Result

Improved model performance. Our approach makes use of the color prioritization effect in attention in order to comply with human perceptual characteristics, which improves the performance of the salient object detection model. We report results for our improved salient object detection model (RNCw) with its base models (RC). Table 1 shows the MAE and S-measure scores on two datasets. We can see that the RNCw model can predict salient regions in complex scenes and distinguish the non-salient regions in the scene. That is, compared with the RC model, the salient regions of our saliency maps are more prominent, and the non-salient regions are rarely mistakenly predicted. On the ECSSD and PASCAL datasets, we obtained the same analysis results. Although our improved algorithm is targeted at salient object detection, its performance on human fixed prediction benchmarks has also improved (see Table 2). In addition, a large number of experimental results show that the salient object detection model (RNCw) based on priori color perception designed in this paper can improve the performance and efficiency of saliency detection to some extent.

**TABLE 1:** The S-measure and MAE on two salient object detection datasets.

Metric	Dataset	RC	RNCw
MAE	ECSSD	0.188	0.173
	PASCAL-S	0.300	0.289
S-measure	ECSSD	0.651	0.669
	PASCAL-S	0.584	0.589

**TABLE 2:** The ROC areas on three eye-tracking datasets.

Method	CGed	CAT2000	OSIE
RC	0.7131	0.7233	0.7310
RNCw	0.7251	0.7294	0.7408

### 6.2 Fixation prediction

#### 6.2.1 Datasets

In addition to the CGed dataset, we tested APNet on two other eye-tracking datasets. One is the CAT2000 training set, which contains 2,000 images, and another is the OSIE dataset, which contains 700 images.

#### 6.2.2 DNN parameters

We train our APNet by first initializing the weights and biases from the VGG-16 model on ImageNet. We use a momentum of 0.9 and a weight decay of 0.0005. The learning rate is 0.00005 and the batch size is 32. The entire training procedure takes about 1 day to complete on a single NVIDIA V100 GPU using the caffe deep learning framework Sharma et al. (2015).

#### 6.2.3 Evaluation metrics

We use nine metrics for comprehensive evaluation. The Area Under the Curve (AUC) Green and Swets (1966) is the area under a curve of true positive rate versus false positive rate for different thresholds on the saliency map. We use two variants of AUC: AUC-Judd and AUC-Borji Bylinskii et al. (2017), and shuffled-AUC (sAUC) Tatler et al. (2005), which alleviates the effects of center bias. The Normalized Scanpath Saliency (NSS) Peters et al. (2005) computes the average value at all fixations in a normalized saliency map. Similarity (SIM) Judd et al. (2012) calculates the sum of minimum values of saliency distribution and fixation distribution at each point. The saliency map can be compared with the human fixation map with the Linear Correlation Coefficient (CC) Le Meur et al. (2007) and the Kullback-Leibler divergence (KL) Tatler et al. (2005). The Earth Movers Distance (EMD) Rubner et al. (2000) considers the ground-truth and predicted saliency maps to be two probability distributions and measure the cost of transferring one distribution to the other. Information Gain (IG) Bylinskii et al. (2017) as an information theoretic metric that measures saliency model performance beyond systematic bias (e.g., a center prior baseline).

#### 6.2.4 Result

**FIGURE 5:**
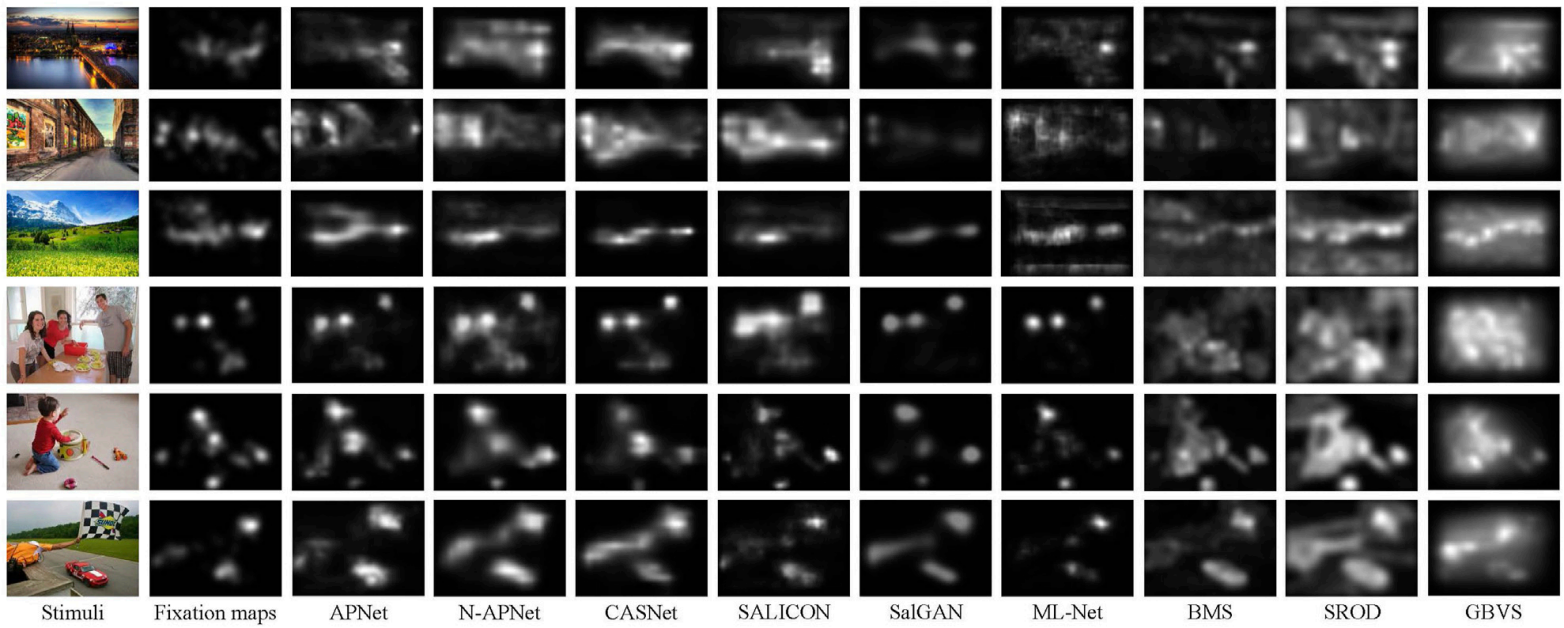
Visual comparisons of results generated by our saliency model with state-of-art methods. Our model (APNet) outperforms others in both location and order, by taking into consideration color prioritization effects in attention allocation within an image.

Tables 3–5 give the quantitative results of comparison with state-of-the arts models on OSIE dataset, CAT2000 dataset and CGed dataset respectively, and Figure 5 shows visual comparisons of results generated by our saliency model with previous methods. We report results for our model both with the subnetwork for context saliency prediction (APNet) and without the subnetwork (N-APNet—Not Adaptive color weighting and priori attention weighting Network). We compared saliency prediction models with seven others. There are state-of-the-art DNN-based models: CASNet Fan et al. (2018), SALICON Thomas (2016) (We use the code of OpenSALICON Thomas (2016) which is a publicly available implementation of SALICON), SalGAN Pan et al. (2017), and ML-Net Cornia et al. (2016). Four are non-DNN models with top performance in the non-DNN model category: BMS), SROD, and GBVS. These models achieved state of the art performance in their experiments on benchmark.

**TABLE 3:** Quantitative results on CGed dataset (color images). The best performance in each metric is highlighted in bold. For all evaluation metrics larger values indicate performance, except smaller is better for EMD and KL.

Metric	APNet	N-APNet	CASNet	SALICON	SalGAN	ML-Net	BMS	SROD	GBVS
AUC-Judd	**0.83**	0.82	**0.83**	0.81	0.82	0.80	0.77	0.75	0.71
AUC-Borji	**0.82**	0.81	0.80	0.79	0.77	0.77	0.76	0.74	0.70
sAUC	**0.74**	0.73	0.73	0.72	0.73	0.71	0.72	0.70	0.66
NSS	**1.76**	1.72	1.75	1.71	1.73	1.72	1.44	1.35	1.11
IG	**6.41**	6.37	6.39	6.31	6.40	6.37	6.24	6.31	6.01
CC	**0.74**	0.72	**0.74**	0.73	0.73	0.72	0.54	0.52	0.40
SIM	**0.62**	0.58	**0.62**	0.57	0.58	0.58	0.53	0.52	0.44
EMD	**4.12**	4.17	4.15	**4.12**	4.17	4.23	6.31	6.33	6.57
KL	**5.99**	6.09	6.05	6.13	6.21	6.24	6.34	5.37	6.44

**TABLE 4:** Quantitative results on CAT2000 dataset. The best performance in each metric is highlighted in bold.

Metric	APNet	N-APNet	CASNet	SALICON	SalGAN	ML-Net	BMS	SROD	GBVS
AUC-Judd	**0.82**	0.81	0.82	0.80	0.81	0.79	0.78	0.77	0.80
AUC-Borji	**0.80**	0.77	0.79	0.78	0.80	0.73	0.77	0.76	0.79
sAUC	**0.79**	0.74	0.76	0.77	0.77	0.75	0.73	0.72	0.75
NSS	**1.51**	1.36	1.50	1.45	1.45	1.35	1.15	1.07	1.25
IG	**0.37**	0.25	**0.37**	0.09	0.08	0.27	-0.17	-0.21	-0.25
CC	**0.59**	0.52	0.58	0.56	0.56	0.52	0.44	0.41	0.49
SIM	0.55	0.53	**0.57**	0.53	0.53	0.52	0.49	0.48	0.51
EMD	2.86	2.89	**2.42**	3.21	3.21	2.86	3.12	3.31	3.12
KL	**5.77**	5.84	5.82	6.03	6.08	6.08	6.21	6.06	6.29

**TABLE 5:** Quantitative results on OSIE dataset. The best performance in each metric is highlighted in bold.

Metric	APNet	N-APNet	CASNet	SALICON	SalGAN	ML-Net	BMS	SROD	GBVS
AUC-Judd	**0.88**	0.87	**0.88**	0.87	0.87	0.86	0.84	0.81	0.78
AUC-Borji	**0.86**	0.83	0.85	0.84	0.84	0.78	0.81	0.80	0.73
sAUC	0.84	0.74	**0.85**	0.82	0.82	0.77	0.79	0.78	0.73
NSS	**2.38**	2.37	2.36	2.31	2.26	2.37	1.54	1.33	0.37
IG	**2.99**	2.91	2.89	2.93	2.84	2.77	2.43	2.18	2.34
CC	0.72	0.69	**0.75**	0.69	0.72	0.72	0.48	0.43	0.44
SIM	0.57	0.54	**0.59**	0.53	0.60	0.61	0.43	0.40	0.42
EMD	2.99	3.23	2.97	3.21	2.94	**2.78**	4.10	4.33	4.42
KL	**5.80**	5.83	5.82	5.90	5.91	5.91	6.30	6.48	6.44

The comparison method ensures fairness. We exclude DNN models that use or learn center bias (e.g., SAM-ResNet Cornia et al. (2018)). All DNN-based models are trained on the SALICON dataset to achieve their best possible performance, and all models, including ours, are directly tested on the three benchmark datasets without training/fine-tuning on them.

**FIGURE 6:**
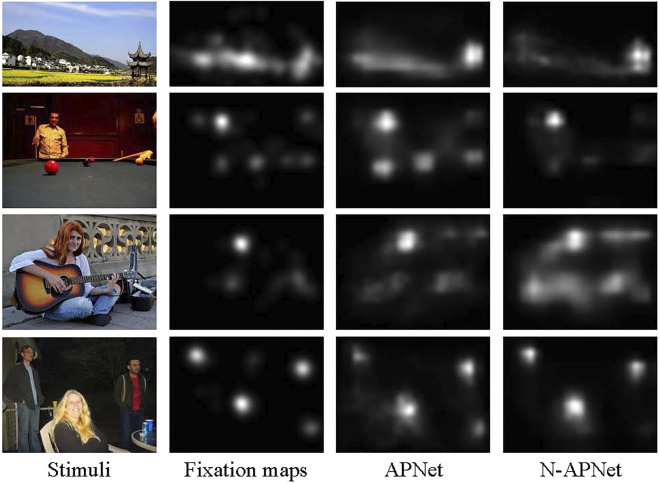
APNet outperforms N-APNet in colorful scenes. APNet uses contextual information to improve fixation prediction by learning the relative importance of colorful objects. Compared to N-APNet, APNet’s predictions more closely match human color perception priorities.

As shown in the quantitative results, the proposed APNet model with the contextual saliency subnetwork has the best overall performance across dataset, without additional center bias mechanism. APNet consistently outperforms N-APNet on all datasets, indicating the effectiveness of learning the relative weights of salient regions inside an image through the proposed subnetwork. On AUC-judd, NSS, IG, and KL, APNet consistently outperforms. For other metrics, APNet is not always the best, but it is close to the best. As we all know, NSS and IG consider the relative importance of salient regions and are therefore the best evaluation measures for contextual saliency. APNet beats the other models on these two metrics across all three datasets, demonstrating its advantage in contextual saliency. In Figure 6, we analyze the effectiveness of APNet in learning the relative importance of contextual information for brightly colored objects.

## 7 Conclusion

Studies conducted in the paper focus on understanding how humans perceive and prioritize colors in scenes and how it is related to visual attention. Based on the statistical results on a collected CGed dataset, we proposed a novel salient object detection model and a novel fixation prediction model considering the factor of color computationally. Our current work is still limited in dataset size, and the study of colorized effects on visual saliency still needs further exploration. For future work, we will collect more data to cover different scenes and different subjects. Previous work showed that fusing multi-scale features Huang et al. (2022) or multi-modal information such as depth Jiang et al. (2021) is useful, which could be another future direction. Moreover, considering that the fixation prediction models are constructed originally to understand human visual attention and eye movement prediction, while the saliency detection models were driven by the requirements of saliency-based applications, another future work could focus on integrating the two tasks of eye fixation prediction and salient object detection, to enhance the performance of both types of models. The proposed color-aware saliency computing methods can also be extended to benefit other related areas such as object proposal generation and segmentation.

## Data Availability

The raw data supporting the conclusion of this article will be made available by the authors without undue reservation, upon request.
